# Evaluation of Strategies for Measuring Lysosomal Glucocerebrosidase Activity

**DOI:** 10.1002/mds.28815

**Published:** 2021-10-06

**Authors:** Daniel Ysselstein, Tiffany J. Young, Maria Nguyen, Shalini Padmanabhan, Warren D. Hirst, Nicolas Dzamko, Dimitri Krainc

**Affiliations:** ^1^ Vanqua Bio Chicago Illinois USA; ^2^ Ken and Ruth Davee Department of Neurology Northwestern University Feinberg School of Medicine Chicago Illinois USA; ^3^ The Michael J. Fox Foundation for Parkinson's Research New York New York USA; ^4^ Neurodegenerative Diseases Research Unit Biogen Cambridge Massachusetts USA; ^5^ Brain and Mind Centre and Faculty of Medicine and Health, School of Medical Sciences University of Sydney Camperdown New South Wales Australia

**Keywords:** glucocerebrosidase, GCase, GCase enzyme activity, Parkinson's disease

## Abstract

Mutations in *GBA1*, which encode for the protein glucocerebrosidase (GCase), are the most common genetic risk factor for Parkinson's disease and dementia with Lewy bodies. In addition, growing evidence now suggests that the loss of GCase activity is also involved in onset of all forms of Parkinson's disease, dementia with Lewy bodies, and other dementias, such as progranulin‐linked frontal temporal dementia. As a result, there is significant interest in developing GCase‐targeted therapies that have the potential to stop or slow progression of these diseases. Despite this interest in GCase as a therapeutic target, there is significant inconsistency in the methodology for measuring GCase enzymatic activity in disease‐modeling systems and patient populations, which could hinder progress in developing GCase therapies. In this review, we discuss the different strategies that have been developed to assess GCase activity and highlight the specific strengths and weaknesses of these approaches as well as the gaps that remain. We also discuss the current and potential role of these different methodologies in preclinical and clinical development of GCase‐targeted therapies. © 2021 The Authors. *Movement Disorders* published by Wiley Periodicals LLC on behalf of International Parkinson and Movement Disorder Society

Glucosylceramidases are a family of enzymes encoded by the genes *GBA1*, *GBA2*, and *GBA3* that play an important role in maintaining cellular homeostasis via the metabolism of glucosylceramide to ceramide and glucose. Glucosylceramidase β, better known as glucocerebrosidase (GCase), is encoded by *GBA1*, ubiquitously expressed and predominantly localized in the lysosome.^1^ Glucosylceramidase β 2, encoded by *GBA2*, is also ubiquitously expressed but localized in the cytoplasm.^2^ Consequently, the enzymes encoded by *GBA1* and *GBA2* are also often referred to as lysosomal and nonlysosomal glucosylceramidase, respectively. For the purpose of this review, however, the enzymes encoded by *GBA1* and *GBA2* are referred to as GCase and GBA2, respectively. These two enzymes show little sequence homology to each other. However, they still share overlapping substrate specificity, with GCase and GBA2 metabolizing substrates at a different pH due to the different lysosomal/cytoplasmic intracellular locations. Glucosylceramidase β 3, encoded by *GBA3*, is also cytoplasmic, but with an expression restricted to the liver and with seemingly much less affinity to metabolize glucosylceramide.^3^ Due to its role in human disease, the majority of studies to date have focused on lysosomal GCase, encoded by *GBA1*. GCase is synthesized in the endoplasmic reticulum (ER) and contains 497 amino acids, including a signal peptide that is cleaved off to produce the mature protein. In the ER, GCase acquires 4 N‐linked glycans^4^ and is complexed with lysosomal integral membrane protein‐2 (LIMP‐2), which is encoded by the *SCARB2* gene. The LIMP‐2‐GCase complex is transported to the Golgi where additional glycosylation occurs. Once in the acidic late endosome/lysosomal compartments, the complex dissociates and GCase then interacts with saposin C, which is a protein cofactor for GCase activity. In addition to metabolism of glucosylceramide, lysosomal GCase can also hydrolyze glucosylsphingosine, although this occurs at a much slower rate.

Homozygous or compound heterozygous *GBA1* mutations lead to development of the lysosomal storage disorder, Gaucher disease (GD). More than 400 mutations in *GBA1* have been associated with this disease,^5^ including point mutations, splice‐site mutations, deletions, insertions, and aberrant recombination that result in either disrupted translation, misfolding, impaired trafficking, reduced enzyme stability, reduced enzymatic efficiency, or a combination of these defects. Different *GBA1* mutation types may underlie the development of the different types of GD (type 1, type 2, or type 3), which differ in severity and the manifestation of clinical symptoms. Regardless of the mutation type, however, the end result is a significant impairment in GCase enzyme function in the lysosome, resulting in the progressive accumulation of glucosylceramide, particularly in the cells of the mononuclear phagocyte system. These cells are transformed into Gaucher cells, which have a distinct enlarged lipid‐laden macrophage phenotype.^6^ In addition, accumulating glucosylceramide in the lysosome can be converted to glucosylsphingosine by the lysosomal enzyme acid ceramidase.^7^ Glucosylsphingosine is more hydrophilic than glycosylceramide, which is thought to allow its escape from the lysosome^7^ and contribute to toxicity in GD.^8^ In severe GD, glucosylceramide also accumulates in the central nervous system (CNS), predominantly in perivascular macrophages,^9^ but also in neurons,^10^, ^11^, ^12^ which is thought to promote the neuroinflammation observed in GD.^10^

Subsequent clinical and genetic sequencing analyses revealed that heterozygous mutations in *GBA1* are a major risk factor for the neurodegenerative diseases Parkinson's disease (PD) and dementia with Lewy bodies (DLB), with predicted frequencies of 7% to 12% in patient populations of both PD and DLB.^13^, ^14^, ^15^, ^16^ In a key early study, the reduction in lysosomal GCase activity resulted in the accumulation of glucosylceramide that stabilized toxic α‐synuclein oligomers. This study also found that the accumulation of α‐synuclein interferes with ER to Golgi trafficking of GCase, leading to the formation of a positive feedback loop that, after a threshold, leads to self‐propagating disease regardless of whether there is a mutation in *GBA1*.^17^ Subsequent studies have also demonstrated a reduction in wild‐type (WT) GCase activity in patient blood samples,^18^ cerebrospinal fluid (CSF),^19^ and postmortem brain tissue^20^, ^21^, ^22^ highlighting a potential role for GCase in the pathogenesis of sporadic and familial forms of PD. Studies in induced pluripotent stem cell (iPSC)–derived dopaminergic neurons from patients with PD showed that either α‐synuclein or oxidized dopamine could lower WT GCase activity in genetic or idiopathic forms of PD.^17^, ^23^ Another recent study also described a reduction in GCase activity in idiopathic PD fibroblast driven by reduced LIMP‐2 expression.^24^ Collectively, these studies highlight decreases in GCase activity as an important contributor of PD pathogenesis and provide rationale for further studying the upstream regulators of GCase activity to develop additional novel strategies to target this protein in PD.

In GD, visceral symptoms are markedly improved by enzyme replacement therapy through chronic intravenous administration, which results in enzyme uptake by affected macrophages. However, the inability of the infused recombinant enzyme to pass through the blood–brain barrier prevents this approach from affecting the neurological manifestation of GCase deficiency observed in PD or DLB. As a result, various strategies have been developed to restore or replace GCase activity in the brain for PD and neuronopathic GD. Small molecule therapeutics currently under development include molecular chaperones and positive allosteric modulators (Table 1). The goal of molecular chaperones is to assist in the folding of mutant GCase in the ER, thereby improving trafficking from the ER to the lysosome and/or increasing the stability of the resulting lysosomal enzyme to improve protein longevity and accumulation of active protein in the lysosome. The goal of positive allosteric modulators is to pharmacologically increase the enzymatic efficiency of WT lysosomal GCase to compensate for activity lost by a heterozygous mutation. Other therapies that are in development or being tested include gene therapy to express WT GCase, linking recombinant GCase to a protein shuttle to enable active transport of enzyme into the brain, and CRISPR‐based approaches to correct mutations in the *GBA1* gene (Table 1).

**TABLE 1:** Current/proposed therapeutic strategies targeting GCase

Therapeutic strategy	Example	Phase in drug development	Summary of results	GCase activity measurement technique
Molecular chaperone	Ambroxol	Phase II completed	Decreased CSF GCase activity and increased protein levels	4‐MUG in vitro
Activator	BIA 28‐6156/LTI‐291	Phase I completed	Effects on GCase activity not publicly disclosed	N/A
Gene therapy	PR001	Phase I/II ongoing	CSF GCase activity increased from undetectable to within normal range	N/A
Transport vehicle modified recombinant GCase	ETV:GBA	Preclinical research ongoing	No current publications	N/A

Abbreviations: GCase, glucocerebrosidase; CSF, cerebrospinal fluid; 4‐MUG, 4‐methylumbelliferyl‐β‐D‐glucopyranoside; ETV, enzyme transport vehicle.

With different treatment modalities being tested preclinically and clinically, robust assays are required to measure the levels and activity of GCase so the effect of GBA‐targeted therapies can be accurately assessed. These assays could also play a critical role in patient inclusion criteria for clinical trials. GCase activity can vary widely in the patient population, even in patients with GCase mutations. Therefore, the ability to identify patients with low GCase activity may be a way to select patients who are more likely to respond to GCase‐targeted therapy. This selection could increase the likelihood of success of new therapeutics and also ensure that future therapies are targeted to relevant patient populations. Despite the considerable advances in assay technologies, there is significant inconsistency in the methodology for measuring GCase activity in disease‐modeling systems and patient populations. Hence, there is a critical need for uniform recognition of the strengths and weaknesses of these various approaches. Such an understanding is crucial for further development of strategies to measure target engagement of novel therapeutics for GCase.

Here we discuss different approaches that have been used to assess GCase activity as well as the potential roles of these measurements in the development/evaluation of new therapeutics (Table 2). The specific approaches discussed were selected because they are the most widely used in the field and most relevant to preclinical development. It is important to note that although each of these assays provide information on the function of GCase, they only serve as artificial proxies of the cellular function of GCase, which is the metabolic turnover of glycosylceramide and glucosylsphingosine in the lysosome. Therefore, the ultimate effect of GCase‐targeted therapies should be reliably measured through lipidomic‐based analyses. This has been done in peripheral blood mononuclear cells (PBMCs), serum, and CSF, although for serum and CSF measurements it is unclear how accurately these levels reflect what is occurring in the lysosome.

**TABLE 2:** Summary of commonly used strategies for assessing lysosomal GCase

Assay	Substrate examples	Measures	Best applications	Application for therapeutic development	Disadvantages
Recombinant protein in vitro activity	4‐MUG, ResGlu, BODIPY glucosylceramide	GCase activity of recombinant protein	Analyzing direct effects of different environments/compounds on GCase enzyme kinetics	High‐throughput screening for GCase activators; confirming lack of inhibitory activity for chaperones	Does not account for variation in endogenous lysosomal factors that can affect activity
Cell lysate in vitro activity	4‐MUG, ResGlu BODIPY glucosylceramide	Total GCase protein that includes lysosomal and nonlysosomal GCase	Analyzing total GCase protein, the effect of GCase mutations and covalent modification on GCase activity	Proof‐of‐concept studies for GCase chaperones and gene therapies	Is not able to correct for difference in GCase levels, which affect measured activity
Patient biofluid in vitro activity	4‐MUG, ResGlu	Total GCase protein	Activity measurement in serum and CSF	Evaluation of target engagement, patient selection	Function of GCase in serum and CSF and correlation with tissue activity is unknown
Western blotting	Antibody	Total GCase protein, ER GCase, post‐ER GCase	Quantifying ER retention of GCase and post‐ER GCase	Proof‐of‐concept studies for GCase chaperones and gene therapies	Does not report on enzyme activity
Inhibody	MDW333, MDW941	Lysosomal GCase protein	Quantifying lysosomal GCase protein, analyzing GCase protein by microscopy	Proof‐of‐concept studies for GCase chaperones and gene therapies	Quantifies levels of active protein not enzyme activity
In situ GCase activity—cell culture	PFB‐FDGlu	In situ lysosomal GCase activity	Analyzing lysosomal GCase activity while accounting for endogenous factors	Screening, proof‐of‐concept studies for GCase chaperons, gene therapies, and activators	Measurement will be affected by differences in substrate uptake
In situ GCase activity—PBMC	PFB‐FDGlu	In situ lysosomal GCase activity	Analyzing lysosomal GCase activity while accounting for endogenous factors	Verify target engagement of chaperones and activators, patient selection	Measurement will be affected by differences in substrate uptake
Dry blood spot assay	C12 glucosylceramide	Total GCase protein that includes lysosomal and nonlysosomal GCase	Analyzing total GCase protein, the effect of GCase mutations, covalent modification on GCase activity	Patient selection, target engagement of GCase chaperones	Requires specialized sample preparation and equipment; does not account for variation in endogenous lysosomal factors

Abbreviations: 4‐MUG, 4‐methylumbelliferyl‐β‐D‐glucopyranoside; ResGlu, Reresorufin‐β‐D‐glucopyranoside; BODIPY, boron dipyrromenthene; GCase, glucocerebrosidase; CSF, cerebrospinal fluid; PFB‐FDGlu, 5‐(Pentafluorobenzoylamino) Fluorescein Di‐β‐D‐Glucopyranoside; ER, endoplasmic reticulum; PBMC, peripheral blood mononuclear cell.

## In Vitro GCase Activity Using Fluorescent Substrates

The most commonly used method to evaluate GCase activity is the use of artificial fluorescent substrates combined with in vitro systems. This technique uses either recombinant GCase protein or protein extracted from cellular model systems including patient fibroblasts and iPSCs as well as animal or patient tissues or biofluids. The protein is then diluted in an acidic enzyme assay buffer to mimic the low pH of the lysosome. A critical component of the assay system is the addition of a lipid or detergent to maintain the enzyme in an active confirmation. This is necessary as delipidate GCase is essentially inactive.^25^ There is significant variation in the lipid/detergent used in literature. The most commonly used is the bile salt taurocholate, however, neutral detergents or the acidic phospholipid phosphatidylserine are also common. To monitor enzymatic activity, several fluorescent probes have been developed. These include the blue fluorogenic substrate 4‐methylumbelliferyl‐β‐D‐glucopyranoside (4‐MUG)^26^ or the red fluorogenic substrate resorufin‐β‐D‐glucopyranoside.^27^ Although the lower pH in the reaction buffer is selective for lysosomal GCase, it is common to simultaneously treat samples with a selective GCase inhibitor such as conduritol B epoxide (CBE), or isofagomine, to determine the background signal in the system and remove any contribution of substrate hydrolysis by GBA2, which can also hydrolyze 4‐MUG, although far less efficiently at lower pH. Alternatively, a GBA2 selective inhibitor such as *N*‐Butyldeoxynojirimycin (NB‐DNJ) could be used to isolate GCase specific activity.^28^ There are a number of factors that are essential to consider when setting up an in vitro GCase assay. The most important is to ensure that the enzyme kinetics are linear at the time of fluorescence measurement. Dilution of GCase into an assay buffer has been shown to reduce the stability of the enzyme. This is particularly important when assessing the activity of mutant enzymes that are less stable than the WT enzyme. Linear kinetics is essential for accurate comparison of GCase activity and should be optimized prior to quantification. Another important consideration is the lysis buffer used to generate the cell/tissue lysates because GCase activity is very sensitive to the presence of detergent; the specific detergent used in the cell or tissue lysis buffer can significantly affect the apparent activity of GCase.

As discussed, significant variation exists in the exact conditions used for in vitro GCase activity assay. Instead of delving into the different buffer systems, pH, and detergents used in published in vitro GCase assays, it is important to determine what the buffering system accomplishes. In any in vitro enzyme assay, the biochemical activity of the enzyme is measured outside a biological system. As a result, this assay does not take into account in situ factors such as variations lysosomal pH, natural allosteric regulators, the presence of cofactors such as saposin C, or the current state of GCase in the ER or the lysosome. In an in vitro assay, the activity measurement is proportional with the total GCase protein in the sample. This limitation is highlighted by the observation that in rare instances of Gaucher‐like disease caused by loss of saposin C, the activity of GCase is normal when measured by an in vitro GCase assay.^29^ Therefore, in this assay design, the only major factor that could influence observed reaction rate is the presence of mutations that affect enzyme function or the presence of covalent posttranslational modifications.^23^ This is why in vitro GCase assays are diagnostic in GD and may help to identify *GBA1* mutation carriers.

### Advantages/Disadvantages

The use of in vitro GCase activity assays has had a significant impact on GCase research and therapeutic development. These assays have been used to diagnose GD and evaluate GCase activity derived from the tissue of patients with PD. Because of the robust assay signal, these approaches have been successfully used in high‐throughput screening.^30^ In addition, when evaluating the efficacy of molecular chaperones or gene therapy, the resulting increase in GCase protein can be detected using these strategies. Lastly, a major advantage of this approach is that is allows for the absolute quantification of GCase enzyme activity. In cell/tissue lysates, this is expressed as nmol/mg protein/h; in patient fluids, this is expressed as μmol/L/h. In theory, this enables comparisons across different studies in the literature; however, this is only possible if identical assay conditions are used, which is rarely the case.

**FIG 1:**
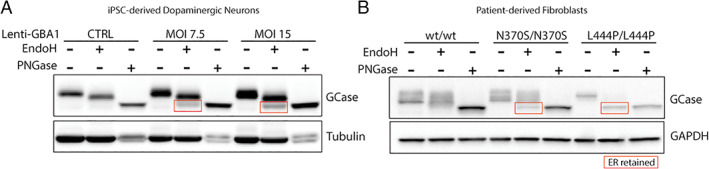
ER‐retention of GCase in neurons overexpressing *GBA1* and in fibroblasts from a patient with Gaucher disease. (**A**) Western blot analysis of lysates treated with endo H, PNGase F, or untreated from patient‐derived dopaminergic neurons after lentiviral‐mediated over expression of *GBA1* for 2 weeks at MOI of 7.5 or 15 and control neurons treated with lentivirus‐expressing GFP. (**B**) Western blot analysis of lysates treated with Endo H, PNGase F, or untreated from fibroblasts for control or patients with Gaucher disease type I (N370S/N370S) or type II (L444/L444P). CTRL, control; Endo H, Endoglycosidase H; ER, endoplasmic reticulum; GCase, glucocerebrosidase; GFP, green fluorescent protein; iPSC, induced pluripotent stem cell; MOI, multiplicity of infection; wt, wild type.

A major limitation of this assay is that it does not account for endogenous factors that could influence GCase activity. These include mutations in lysosomal enzymes, chemical agents that cause lysosomal dysfunction, or agents that increase lysosomal pH that can lead to the accumulation and enlargement of lysosomes. The effects of these endogenous factors may display as normal or increased levels of GCase activity when using in vitro assays but may significantly alter the in situ GCase activity. An additional concern with this approach is that it does not exclude GCase located in the ER. As we have seen with certain GCase mutations and the overexpression of GCase, there is considerable GCase retained in the ER that could also be included using such in vitro analyses (Fig. 1). In vitro GCase assays are also not useful to assess GCase activation in cellular systems treated with putative positive allosteric modulators, as any modulator is likely to be significantly diluted upon cellular lysis and the addition of reaction buffer. Lastly, differences in structure and affinity of the artificial substrates to mutant GCase may not reflect the affinity of the natural substrate.^31^ In recombinant systems, this has been overcome by the use of natural substrate with mass spectrometry^31^ or using boron dipyrromenthene (BODIPY) labeling with high‐performance liquid chromatography.^32^

### Use in Therapeutic Development

The in vitro GCase activity assay has played an important part in determining the role of GCase in the onset of PD. The assay has been used to show reduced GCase activity as a result of *GBA1* mutations in patient‐derived brain tissue. The assay has also been adapted to measure GCase activity in serum and, more recently, was optimized for measuring GCase activity in the CSF.^33^ As discussed previously, in vitro measurements of GCase activity reflect the amount of GCase protein in the sample. As such, this assay is well suited to measure the effects of molecular chaperones. Systemic administration of molecular chaperones would lead to increased GCase in all cell types including blood cells. Such treatments may also lead to increased GCase protein in serum, although the mechanism through which GCase is released into the serum is unclear. Similarly, increased GCase activity in CSF has recently been reported upon administration of ambroxol, a GCase molecular chaperone.^34^ Although CNS administration of gene therapy would limit peripheral measurements, similar effects on GCase in the CSF could be expected from this approach. This was observed in recent data published by Prevail Therapeutics, which demonstrated significantly increased GCase activity in the CSF in a patient following treatment with PR001,^35^ although the patient in this report was homozygous for *GBA1* mutations, which does not reflect most patients with PD and *GBA1*.

## Measurement of GCase Protein by Sodium Dodecyl Sulfate–Polyacrylamide Gel Electrophoresis

Western blot is the most widely used analytical technique to assess specific proteins in a cell or tissue homogenate. A number of commercial antibodies to GCase have been developed with varying degrees of success. One recent analysis of several antibodies used murine neural cells deficient in GCase, which invalidated a surprising number of commercially available antibodies.^36^ This study serves as a key resource for researchers investigating GCase using Western blot techniques and highlights the importance of proper antibody validation.

The glycosylation of GCase creates an additional challenge for Western blot detection of GCase. Early pulse chase studies revealed that GCase is initially glycosylated in the ER by N‐linked high‐mannose‐type oligosaccharides on four of its five putative sites.^4^ When fully glycosylated, this species runs at an apparent molecular weight of 64 kDa and can be completely deglycosylated by Endoglycosidase H treatment. As GCase is transported toward the lysosome, further maturation of these oligosaccharides occurs in the Golgi apparatus, yielding a species with an apparent molecular weight of 69 kDa. The half‐life for this conversion in patient‐derived fibroblasts was found to be 3 hours.^37^ After an additional 48 hours, the glycosylation can be further modified to a species with an apparent molecular weight of 59 kDa, presumably through modification by lysosomal exoglycosidases. Therefore, both the 59 and 69 kDa species represent post‐golgi GCase protein as they are largely resistant to endo H treatment. Treatment with PNGase F, which removes all N‐linked glycosylations, results in species that have the same apparent molecular weight, indicating that the shift in molecular weights is not due to the proteolytic processing of GCase.^37^

The presence of two apparent molecular weight GCase species in the lysosome has generated some confusion. The prevalence of one species over the other appears to vary depending on the cell line or tissue source that is analyzed. Some researchers have incorrectly indicated the lower 59 kDa molecular weight band as ER‐retained GCase, which has led to the conclusion that in the absence of *GBA1* mutations, a significant fraction of cellular GCase is basally retained in the ER. This is unlikely as the half‐life of GCase in the ER is very short and is supported by evidence that knockdown of the GCase transporter, LIMP‐2, which would theoretically cause all GCase protein to be retained in the ER, leads to an almost complete loss of GCase highlighting the speed at which ER‐GCase is degraded. Unlike the WT enzyme, many of the mutations in GCase can lead to its retention in the ER, which can be identified by examining endo H sensitivity (Fig. 1). This has led to speculation that misfolding in the ER could promote ER stress and modification of disease phenotypes. This has been observed in patient‐derived fibroblasts and animal models of GD^38^, ^39^; however, only preliminary studies have shown a connection in PD.^20^ Further studies are required to establish whether ER stress contributes to pathogenesis of GBA‐PD. A major goal of the molecular chaperone strategy is to assist in proper folding of these ER‐retained forms to allow for optimum transport from the ER. This strategy could be beneficial twofold as it reduces the amount of misfolded protein in the ER and potentially increases the amount of GCase in the lysosome.

### Advantages/Disadvantages

Evaluation of the level of ER‐GCase using endo H sensitivity can be an effective strategy to evaluate the potential of molecular chaperones to improve trafficking of mutant GCase to the lysosome. This approach could also serve to evaluate a concern associated with gene therapy which is that excessive GCase overexpression will overwhelm the ability of LIMP‐2 to traffic the protein to the lysosome, leading to an undesired consequence of GCase accumulation in the ER (Fig. 1). A disadvantage of this approach is that there is considerable noise in the Western blotting technique, making it challenging to accurately obtain quantification. This is especially challenging for GCase as glycosylation provides an additional variable that may affect affinity of the primary antibody to its GCase epitope. Therefore, the treatment of all samples with PNGase F can be used to improve the reliability of total GCase quantification by Western blot.

### Use in Therapeutic Development

The cumbersome, low‐throughput, and variable nature of the sodium dodecyl sulfate–polyacrylamide gel electrophoresis (SDS‐PAGE) technique gives this measurement limited usability in translational approaches. However, the ability to measure ER‐retained GCase makes this a critical method to evaluate therapeutic strategies in cellular and animal models. This technique could provide important proof of mechanism in cell and animal models for molecular chaperones that are designed to bind mutant GCase retained in the ER and enable trafficking to the lysosome. In addition, a concern for the development of small molecules that bind GCase is that they could cause structural changes that affect the LIMP‐2 binding site. These molecules would therefore affect the trafficking of GCase resulting in ER accumulation and potentially less lysosomal GCase. A similar concern exists for gene therapy strategies where increased expression of GCase may lead to ER retention by overwhelming the capacity of LIMP‐2 to traffic GCase to the lysosome. These concerns could be alleviated by examining ER‐retained GCase and titrating the level of expression to ensure that ER retention is mitigated.

## Measurement of GCase Using Inhibodies

**FIG 2:**
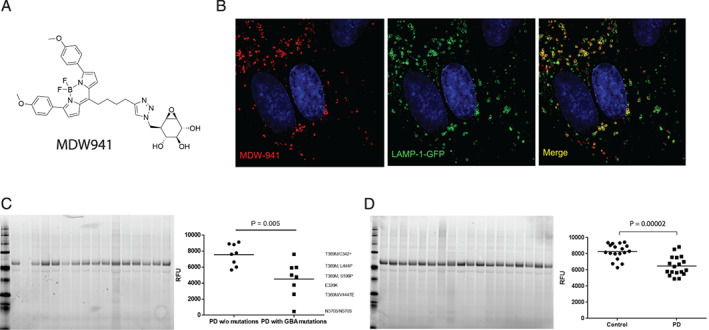
Decreased glucocerebrosidase (GCase) levels in superior temporal gyrus (STG) from *GBA1* mutation carriers and sporadic Parkinson's disease (PD) revealed by Fluorescent GCase Probe. (**A**) Chemical structure of MDW941. (**B**) Representative images from super‐resolution microscopy imaging of cultured human fibroblasts expressing Lamp‐1‐GFP stained with the GCase probe MDW‐941. (**C**) Representative sodium dodecyl sulfate–polyacrylamide gel electrophoresis (SDS‐PAGE) analysis of STG lysates derived from patients with PD with and without *GBA1* mutations treated with MDW‐941. Genotypes for each data point are shown on the right. (**D**) SDS‐PAGE analysis of STG lysates derived from healthy controls or patients with PD without *GBA1* mutations. Data are presented as the mean fluorescence signal from MDW‐941‐modified GCase with individual data points representing unique samples. Data were analyzed using two‐way analysis of variance followed by a Bonferroni post hoc test. GFP, green fluorescent protein; LAMP‐1, lysosome‐asociated membrane protein 1; RFU, relative fluorecent units,

Another approach that has been developed to visualize lysosomal GCase levels is the development of inhibodies.^40^ This approach made use of epoxides such as CBE and cyclophellitol that first bind noncovalently to GCase at the active site and then react with glutamate 340, forming a covalent bond that irreversibly inhibits the enzyme. Fluorescent BODIPY analogs were attached to cyclophellitol using a triazole linker, which led to the generation of MDW333 and MDW941.^40^ These fluorescent probes can be incubated with cultured cells where they react with lysosomal GCase and produce a clear lysosomal staining pattern in live cells that can be analyzed by microscopy (Fig. 2A,B) or flow cytometry. Similarly, the probes can be injected intravenously in mice. After incubation, the level of GCase in tissue lysates can be examined using SDS‐PAGE, although this utility is limited to peripheral tissue as the probe is not able to access GCase in the brain. Recently, this limitation was overcome by directly applying probes to the CNS through intracerebroventricular administration.^41^

### Advantages/Disadvantages

The use of inhibody‐based probes has an advantage over in vitro activity assays and Western blotting as it allows for relative quantification of active GCase protein levels in live cells or tissue lysates (Fig. 2C,D). This enables the use of less‐biased high‐content imaging and flow cytometry–based approaches to quantify GCase levels. This could be especially useful for evaluation of target engagement of molecular chaperone‐based approaches. However, it is unclear what effect lysosomal pH could have on the fluorescent intensity of the BODIPY fluorophore, as this would have implications for quantification. Although the probes were shown to be predominantly active at lower pH, they retain modest inhibitory activity at neutral pH.^40^ Therefore, it is unclear to what extent they will react with ER‐retained GCase, although preliminary data show strong lysosomal localization of the probe in treated cells (Fig. 2B). Lastly, although these probes do label GCase in live cells, they have similar limitations as the in vitro GCase activity measurements as they will only measure the total amount of GCase in the lysosome and not account for endogenous lysosomal conditions that could affect GCase activity.

## Measurement of In Situ GCase Activity Using PFB‐FDGlu

**FIG 3:**
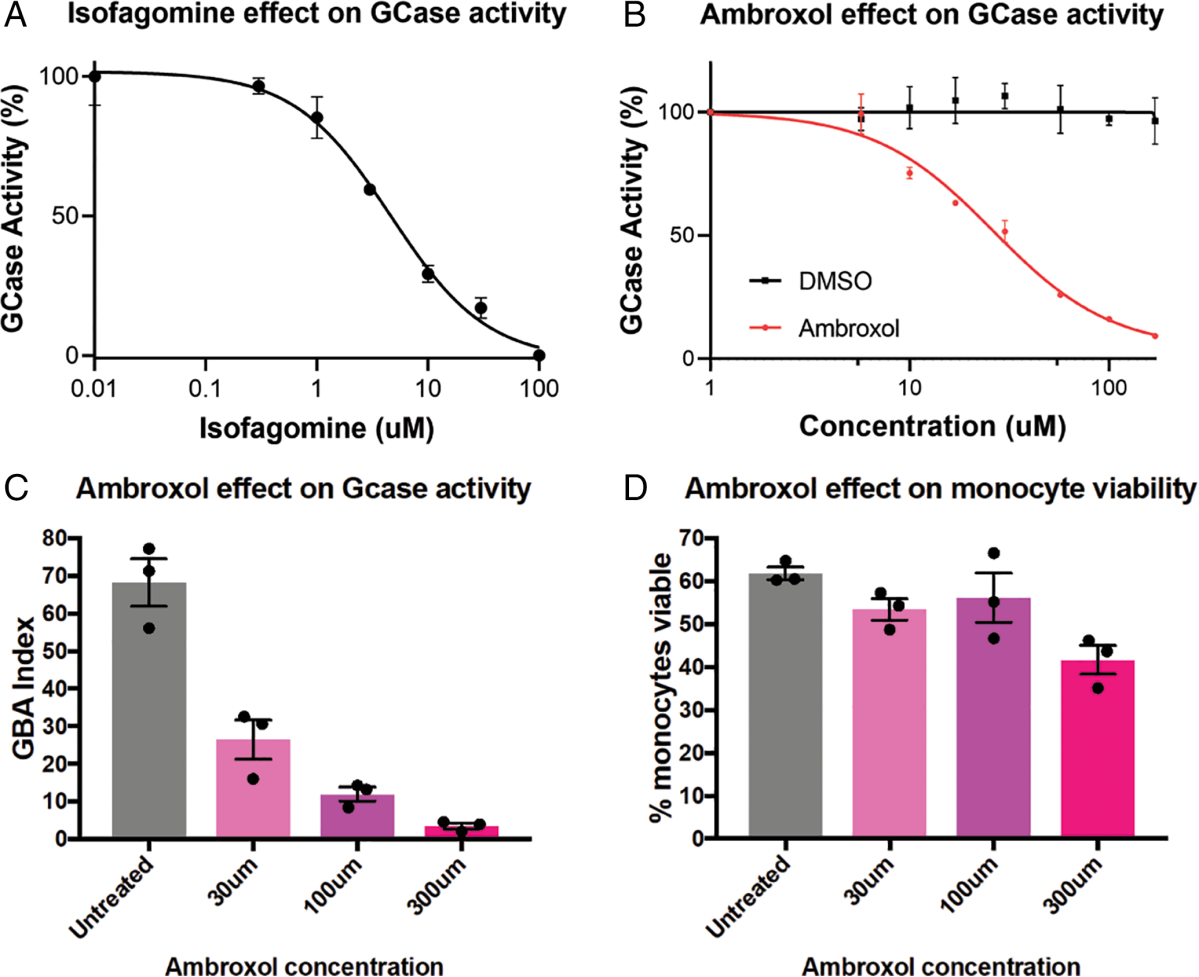
Dose‐dependent reduction in live‐cell GCase activity in the presence of isofagomine or the GCase chaperone ambroxol. (**A**, **B**) Dose‐response curve showing inhibition of lysosomal GCase activity by isofagomine (**A**) or by ambroxol (**B**) in cultured HeLa cells. (**C**) GCase activity measured in CD14‐positive peripheral blood‐derived monocytes treated with increasing concentrations of ambroxol. (**D**) Evaluation of the effect of ambroxol on monocyte viability. The data are presented as a GBA activity index, which is the ratio of 5‐(Pentafluorobenzoylamino) Fluorescein Di‐β‐D‐Glucopyranoside (PFB‐FDglu) signal without conduritol B epoxide (CBE) divided by the PFB‐FDglu signal with CBE. Data were analyzed by one‐way analysis of variance and Dunnett's multiple comparison test. *P < 0.05 compared with the untreated group. Graphs show mean ± standard error of the mean, with the dots representing individual data points. DMSO, dimethylsulfoxide; GCase, glucocerebrosidase.

As mentioned previously, a major disadvantage of in vitro assays to measure GCase activity is that they do not account for changes in the lysosomal microenviroment that could impact GCase activity. To overcome this limitation, the cell permeable GCase substrate 5‐(Pentafluorobenzoylamino) Fluorescein Di‐β‐D‐Glucopyranoside (PFB‐FDGlu) can be used. PFB‐FDGlu is a fluorescent‐quenched probe that yields green fluorescence upon hydrolysis by GCase. The probe is taken up in the cell by pinocytosis and trafficked through the endosomal system to the lysosome where it can be cleaved by lysosomal GCase.^42^ To correct for background fluorescence and potential off‐target hydrolysis of PFB‐FDGlu by cytosolic GCase, cells can be incubated with the GCase selective inhibitors CBE or isofagomine (Fig. 3A).

The PFB‐FDGlu approach has been used to measure in situ GCase activity in a number of cell types including patient‐derived fibroblasts^43^ and liver cells.^44^, ^45^ Recently, PFB‐FDGlu was used to examine GCase activity in iPSC‐derived dopaminergic neurons using a microplate format.^46^ In this study, it was found that mutations in Leucine Rich Repeat Kinase 2 (*LRRK2*) affect GCase activity, despite not influencing the absolute level of GCase protein. In another recent study, where PFB‐FDGlu was used to measure in situ GCase activity in PBMCs from patients with PD, the authors found that when correcting for protein content, monocytes from patients with PD display reduced GCase activity.^47^ Interestingly, although the raw GCase activity in these cells displayed a trend toward reduced activity, analysis of the protein content revealed an increase in GCase levels.^47^ This deviation further highlights the disconnect between in situ GCase activity and GCase protein levels and underscores the importance of considering in situ activity when evaluating GCase activity.

### Advantages/Disadvantages

The advantage of this approach is that it allows for the measurement of in situ GCase activity, which is most relevant to lysosomal function. It also accounts for changes in the lysosomal microenvironment, such as changes in pH, ion content, lipid content, accumulation of misfolded protein, and other factors that have been shown to affect the function of lysosomal enzymes. Evaluation of in situ GCase activity will allow for the expansion of studies on GCase regulation in the lysosome, which could lead to the identification of new therapeutic targets to enhance GCase activity independently of the protein. This potential is highlighted by the identification that LRRK2 kinase inhibitors were found to increase GCase activity in neurons.^46^

However, a weakness of the PFB‐FDGlu approach is that the substrate requires uptake by pinocytosis, which leads to several concerns that must be considered when evaluating relative enzyme activity. As with any enzymatic assay, the rate of hydrolysis of PFB‐FDGlu is dependent on substrate concentration.^42^ Genetic or chemical perturbations that affect the pinocytosis pathway could lead to reduced loading of substate, which may falsely produce differences in GCase activity readout. This also applies in the evaluation of different cell types as the rate of pinocytosis could vary greatly between different cells, leading to artifacts of apparent differences in GCase activity but may simply reflect the differences in pinocytosis rates.

### Use in Therapeutic Development

In situ GCase activity is the most accurate measurement of GCase activity occurring in the lysosome. For this reason, the use of in situ measurements is well suited to evaluate the effects of all therapeutic strategies targeting GCase in cell culture models. This is especially important in the identification of GCase chaperones, as molecular chaperones can often inhibit enzyme activity at elevated concentrations.^48^ This inhibitory effect is observed for ambroxol at micromolar concentrations in cell culture models (Fig. 3B–D). For preclinical animal models, the PFB‐FDGlu assay is more limited. The ability to measure GCase activity in PBMCs would allow the measurement of target engagement for both GCase chaperones and activators in blood. However, it is currently not possible to perform in situ measurement in the CNS, limiting the use of this technique for gene therapy approaches that are CNS administered. Therefore, for early clinical trials, the measurement of GCase activity in patient PBMCs could allow the measurement of target engagement for GCase chaperones and activators in blood, although this may not be feasible in multisite studies for logistical reasons. Perhaps the best role of in situ GCase activity measurements for clinical development is in patient selection. Prescreening patients with PD to identify individuals who have significantly reduced GCase activity in the presence or absence of *GBA1* mutations could increase the likelihood of seeing a significant effect of therapeutic intervention. The assumption is that patients with low PBMC GCase activity will also have low activity in the CNS. This has not yet been established but may warrant further investigation given the potential benefits of this approach.

## Measurement of GCase Activity in Dry Blood Spots

Dried blood spot assays are currently being used for the identification of a range of lysosomal storage disorders, including GD.^49^, ^50^ This technique uses blood blotted onto filter paper to enable simple storage and banking of samples for future analysis. Recent iterations of this technique use mass spectrometer–based detection instead of fluorescent detection, which allows for the measurement of multiple lysosomal enzymes concurrently.

The dry blood spot analysis has been applied to assess GCase activity in patients with PD with and without *GBA1* mutations.^18^ In this study, the researchers included a natural substrate C12‐glucosylceramide for the measurement of enzymatic activity. Specifically, they used punches from stored dried blood spots and upon initial extraction in a neutral buffer, samples were then incubated in an acidic assay buffer containing C12‐glucosylceramide. The samples were analyzed by mass spectrometry to measure the hydrolysis of C12‐glucosylceramide. Recently, this dry blood spot assay was used to assess GCase activity in a 3‐year longitudinal study of 1559 samples from the Parkinson's Progression Markers Initiative cohort.^51^ In concurrence with previous studies, this study reported a significant reduction in GCase activity in samples from patients with PD relative to healthy controls.

### Advantage/Disadvantage

The use of dried blood spots to measure enzyme activity is advantageous due to ease of sampling, shipping, and stability of the samples. The use of mass spectrometer–based approaches is also advantageous as it allows for concurrent measurement of multiple lysosomal enzymes. In addition, this method examines hydrolysis of a natural substrate mimic, C12 glucosylceramide, which avoids concerns associated with artificial substrates, as discussed previously.^31^ A disadvantage of this approach is that there is that more advanced instrumentation is required in contrast to the quick, fluorescence‐based detection methods. In addition, this approach cannot easily account for sampling differences in cell types that may change dramatically from day to day or may exist in a disease population. Although the recent study was able to correct for white blood cell count, future studies could focus on further refinement to specifically account for different cell populations.

### Use in Therapeutic Development

The dry blood spot assay allows for very simple sample collection and storage. This makes the assay well suited to perform longitudinal assessments of GCase activity. As discussed, measurements of GCase activity in dried blood spots are likely to reflect the amount of GCase protein in the sample. As a result, this assay could serve as an excellent strategy to evaluate GCase accumulation resulting from molecular chaperone exposure. This could be applied to preclinical animal studies as well as clinical trials in humans. The ability to collect samples from multiple sites over multiple time points and perform the analysis at a single location is a clear advantage. It may even be possible to adapt this method to measure GCase activity in CSF of individuals treated with chaperones or gene therapy. However, the sample dilution required in this assay would result in dilution of the active compound, therefore, this assay is unlikely to capture effects of GCase activators on enzyme activity.

## Conclusion

There is an increasing recognition that the lysosomal enzyme GCase plays a critical role in the onset of familial and also sporadic PD and DLB. As a result, there is considerable interest in the development of therapies that target GCase to slow or stop progression of these diseases. To enable measurement of GCase activity in disease‐modeling systems and patient populations, a growing number of techniques have been established. This review provides a framework for how these techniques can be used in the preclinical and clinical development of GCase‐targeted therapies.

## Author Roles

(1) Research Project: A. Execution; (2) Manuscript: A. Writing of the First Draft, B. Review and Critique.

D.Y.: 1A, 2A, 2B

T.J.Y.: 1A, 2B

M.N.: 1A, 2B

S.P.: 1A, 2B

W.D.H.: 1A, 2B

N.D.: 1A, 2B

D.K.: 1A, 2B

## Financial Disclosures

D.Y. and M.N. are employees of Vanqua Bio. T.Y. has nothing to declare. N.D. was supported by The Michael J. Fox Foundation and the Shake It Up Australia foundation. W.D.H. is a Biogen employee and shareholder. D.K. is a venture partner with Orbimed Advisors; scientific advisor for Intellia Therapeutics, AcureX, The Silverstein Foundation, and Prevail Therapeutics; and the Founder of Vanqua Bio and Lysosomal Therapeutics. D.K. was supported by National Institutes of Health Grants R01 NS076054, R37 NS096241, R01 NS096240‐01, and U01 NS 094148 and grants from The Michael J. Foundation for Parkinson's Research.

## Supporting information


**Appendix S1**. Supporting Information.

## Data Availability

The data that support the findings of this study are available from the corresponding author upon reasonable request.
