# Supplementary material for: Evaluation of Strategies for Measuring Lysosomal Glucocerebrosidase Activity
Source: Mov Disord. 2021 Oct 6;36(12):2719–30. doi: 10.1002/mds.28815 (PMC8853444; doi:10.1002/mds.28815)
Supplement: Supplementary file 1 — Appendix S1. Supporting Information. [file MDS-36-2719-s001.docx]

Supporting Materials:

**Measurement of GCase activity in Hela Cells:**

Hela cells plated in black 96-well plates were treated with media containing the indicated concentration of Isofagomine, ambroxol, or equivalent amount of DMSO. PFB-FDglu substrate (120 uM) was added to each treatment group in the presence and absence of isofagomine (100 uM). After 4 hours incubation, the cells were washed twice with PBS and fluorescence was measured using the Cytation 5 plate reader. Lysosomal GCase activity is the PFB-FDglu signal with the signal obtained in the isofagomine treated group subtracted. Data is presented as a percentage of GCase activity in untreated cells.

**Evaluation of PNGase and EndoH sensitivity.**

Sensitivity to Endo H and PNGase F was performed according to the manufacturers protocol (New England Biolabs, P0702S, P0704S). A total of 10 μg protein from lysates derived from iPSC-derived dopaminergic neurons or patient-derived fibroblasts were denatured and treated with PNGase (500 units) or Endo H (500 units) in the supplied assay buffer for 1 h at 37°C. Digested lysates were subjected to western blot analysis. GCase that is retained in the ER is sensitive to Endo H treatment and results in a band at equivalent molecular weight to PNGase F treated samples, which removes all glycosylation.

**Measurement of GCase activity in Peripheral blood mononuclear cells:**

Peripheral blood mononuclear cells from healthy donors (n=3) were incubated with the indicated concentrations of ambroxol for 24h. Monocyte GCase activity was measured using the PFB-FDglu substrate probe in the presence and absence of CBE (1mM, 90 min) and gating on viable CD14 positive monocytes. The GBA activity index is the ratio of PFB-FDglu signal without CBE, divided by the PFB-FDglu signal with CBE. Data were analysed by one-way ANOVA and Dunnet’s multiple comparison test. *= p < 0.05 compared to the untreated group. Graphs show mean +/- SEM with the dots representing individual data points.

**Evaluation of lysosomal specificity of MDW941**

Human fibroblasts (“Detroit 551”) (100,000) were plated in Mattek 30mm dishes with glass coverslip bottoms. The next day Organelle Lights-Lysosomes-GFP BacMam 1.0 (Invitrogen) was added overnight. A solution of MDW941 (5nM) and Hoescht was added for 2 hours. Cells were then rinsed 3x with PBS before the addition of phenol red-free growth media. Live imaging of cells was performed on the Zeiss Elyra super-resolution microscope with 100x oil objective. Images were processed with the structured illumination module.

**Measurement of active GCase in human brain tissue lysates**

Human post-mortem superior temporal gyrus samples were homogenized in 10 volumes of GCase lysis buffer containing 0.25% Triton X-100 using Qiagen Tissuelyser at 25 Hz for 4 min x 2. Samples were then sonicated with a Branson Ultrasonics 450 Digital Sonifier (Branson) for 10 seconds at 80 watts at 20 kHz and diluted to 2 mg/mL with lysis buffer. The GCase activity probe MDW941 was added to the samples to a final concentration of 25 nM probe, 5mM citric acid. Lysate and MDW941 probe were incubated at 37 °C for 2 hours. Samples were centrifuged at 21,000 x g for 2 minutes to remove acid precipitates; the supernatant was retained. NuPAGE LDS (4X) sample buffer (ThermoFisher) containing NuPAGE Sample Reducing Agent (dithiothreitol) was added and the mixtures were incubated at 70 °C for 10 min. Protein concentration was determined by BCA (Pierce). 30 µg of protein lysate samples were loaded onto a 4-12% gel NuPAGE (ThermoFisher) with TAMRA labelled fluorescent ladder and probe labelled 5 ng recombinant GCase and run at 150 V in MES buffer until the dye front reached the bottom of the gel. A 20% methanol solution was used to wash away unbound probe. Gels were visualized using a GE Typhoon at 532 nm excitation and 575 nM. Bands were quantified for relative fluorescence units by ImageQuant software (GE). Two-Way ANOVA tests were performed for statistical analysis to understand the effect of genotype. When significance was identified by the Two-Way ANOVA test, Bonferroni *post hoc* tests were used to understand the significance of the individual comparisons. Individual data points, each representing a separate post-mortem STG sample, are represented on each graph.
